# The Promise of Artificial Intelligence in Digestive Healthcare and the Bioethics Challenges It Presents

**DOI:** 10.3390/medicina59040790

**Published:** 2023-04-18

**Authors:** Miguel Mascarenhas, João Afonso, Tiago Ribeiro, Patrícia Andrade, Hélder Cardoso, Guilherme Macedo

**Affiliations:** 1 Faculty of Medicine, University of Porto, 4200-437 Porto, Portugal; 2 Precision Medicine Unit, Department of Gastroenterology, Hospital São João, 4200-437 Porto, Portugal; 3 WGO Training Center, 4200-437 Porto, Portugal

**Keywords:** artificial intelligence, bioethics, medical imaging, big data, gastroenterology, capsule endoscopy, convolutional neural networks, privacy, data protection, bias, responsibility

## Abstract

With modern society well entrenched in the digital area, the use of Artificial Intelligence (AI) to extract useful information from big data has become more commonplace in our daily lives than we perhaps realize. Medical specialties that rely heavily on imaging techniques have become a strong focus for the incorporation of AI tools to aid disease diagnosis and monitoring, yet AI-based tools that can be employed in the clinic are only now beginning to become a reality. However, the potential introduction of these applications raises a number of ethical issues that must be addressed before they can be implemented, among the most important of which are issues related to privacy, data protection, data bias, explainability and responsibility. In this short review, we aim to highlight some of the most important bioethical issues that will have to be addressed if AI solutions are to be successfully incorporated into healthcare protocols, and ideally, before they are put in place. In particular, we contemplate the use of these aids in the field of gastroenterology, focusing particularly on capsule endoscopy and highlighting efforts aimed at resolving the issues associated with their use when available.

## 1. Introduction

Medicine is advancing swiftly into the era of Big Data, particularly through the more widespread use of Electronic Health Records (EHRs) and the digitalization of clinical data, intensifying the demands on informatics solutions in healthcare settings. Like all major advances throughout history, the benefits on offer are associated with new rules of engagement. Some 50 years have passed since what is considered to have been the birth of Artificial Intelligence (AI) at the Dartmouth Summer Research Project [1]. This was an intensive 2-month project that set out to obtain solutions to the problems that are faced when attempting to make a machine that can simulate human intelligence. However, it was not until some years later before the first efforts to design biomedical computing solutions based on AI were seen [2,3,4,5]. These efforts are beginning to bear their fruit, and since the turn of the century, we have witnessed truly significant advances in this field, particularly in terms of medical image analysis [6,7,8,9,10,11,12,13]. Indeed, a search for publications in the PubMed database using the terms “Artificial Intelligence” and “Gastrointestinal Endoscopy” returned 3 articles in 2017, as opposed to 42 in 2022 and 64 in 2021. While the true impact of these practices is yet to be seen in the clinic, their goals are clear: (i) to offer patients more personalized healthcare; (ii) to achieve greater diagnostic/prognostic accuracy; (iii) to reduce human error in clinical practice; and (iv) to reduce the time demands on clinicians as well as enhancing the efficiency of healthcare services. However, the introduction of these tools raises important bioethical issues. Consequently, and before attempting to reap the benefits that they have to offer, it is important to assess how these advances affect patient–clinician relationships [14], what impact they will have on medical decision making, and how these potential improvements in diagnostic accuracy and efficiency will affect the different healthcare systems around the world.

### 1.1. The State-of-the-Art in Gastroenterology

A number of medical specialties such as Gastroenterology rely heavily on medical images to establish disease diagnosis and patient prognosis, as well as to monitor disease progression. Moreover, in more recent times, some such imaging techniques have been adapted so that they can potentially deliver therapeutic interventions [15]. The digitalization of medical imaging has paved the way for important advances in this field, including the design of AI solutions to aid image acquisition and analysis [16,17]. Different endoscopy modalities can be used to visualize and monitor the Gastrointestinal (GI) tract, making this an area in which AI models and applications could play an important future role. Indeed, this is reflected in the attempts to design AI-based tools addressing distinct aspects of these examinations and adapting to the different endoscopy techniques employed in the clinic. Accordingly, the development of such AI tools has been the focus of considerable effort of late, mainly with a view to improving the diagnostic accuracy of GI imaging and streamlining these procedures [18,19]. The term AI is overarching, yet in the context of medical imaging, it can perhaps be more precisely defined by the machine learning (ML) class of AI applications, algorithms that are specifically used to recognize patterns in complex datasets [20]. “Supervised” or “unsupervised” ML models exist; although, the former is perhaps of more interest in this context as they are better suited to attempts at predicting known outputs (e.g., a specific change in a tissue or organ, the presence of a lesion in the mucosa or debris in the tract, etc.). Multi-layered Convolutional Neural Networks (CNNs) are a specific type of deep learning (DL) model, a modality of ML. Significantly, CNNs excel in the analysis, differentiation and classification of medical images and videos, essentially due to their artificial resemblance to neurobiological processes [18,19,20].

As might be expected, there have been significant technical advances in endoscopy over the years. Indeed, two decades have now passed since Capsule Endoscopy (CE: also known as Wireless or Video CE) was shown to be a valid minimally invasive diagnostic tool to visualise the intestine in its entirety, including the small bowel (SB) and colon [21]. CE systems involve the use of three main elements. Firstly, there is the capsule that houses the camera, and now perhaps multiple cameras, as well as a light source, a transmitter and a battery. The second element is a sensor system that is necessary to receive the information transmitted by the capsule and that is connected to a recording system. Finally, there is the software required to display the endoscopy images so they can be examined. All these CE elements have undergone significant improvements since they were initially developed. For example, there have been numerous improvements to the capsules (e.g., in their frame acquisition rates, their angle of vision, the number of cameras, and manoeuvrability), as well as to the software used to visualise and examine the images obtained. One of the benefits of CE is that it offers the possibility of examining less inaccessible regions of the intestine, such as the SB, structures that are difficult to access using standard endoscopy protocols. Consequently, CE can be used to evaluate conditions that are complicated to diagnose clearly, such as chronic GI bleeding, tumours and especially SB tumours; mucosal damage; Crohn’s disease (CD); chronic iron-deficiency anaemia; GI polyposis; or celiac disease [22,23]. There are also fewer contraindications associated with the use of CE; although, these may include disorders of GI motility, GI tract narrowing/obstruction, dysphagia, large GI diverticula or intestinal fistula. Despite the evolution of these systems over the past two decades, they still face a number of challenges, and these will be the target of future improvements.

As indicated, software used to aid in the reading and evaluation of the images acquired by CE has also been developed, on the whole, through efforts to decrease the reading times associated with these tests and the accuracy of the results obtained. The time that trained gastroenterologists must dedicate to the analysis of CE examinations is a particularly critical issue, given the number of images generated (ca. 50,000). As such, considerable effort is required to ensure adequate diagnostic yields, with the high associated costs. Accordingly, the main limitation for CE, and particularly Colon Capsule Endoscopy (CCE), as a first-line procedure for the panendoscopic analysis of the entire GI mucosa, is that it is a relatively time-consuming and laborious diagnostic test that requires some expertise in image analysis. In fact, the diagnostic yield for CE is in part hampered by the monotonous and laborious human CE video analysis, which translates into suboptimal diagnostic accuracy, particularly in terms of sensitivity and negative predictive value (NPV). It must also be considered that alterations may only be evident in a few of the frames extracted from CE examinations, which means there is a significant chance that important lesions might be overlooked [24]. Indeed, the inter- and intra-operator error associated with the reading process is one of the main sources of error in these examinations. As a result, there has been much interest from an early stage in the development of these systems to design software that can be used to automatically detect certain features in the images obtained. For example, there have been attempts to include support vector machines (SVMs) within CE systems, in particular for the detection of blood/hematic traces [25]. In this sense, one of the most interesting recent and future developments in CE is the possible incorporation of AI algorithms to automate the detection, differentiation and stratification of specific features of the GI images obtained [26,27].

### 1.2. Automated Analysis and AI Tools to Examine the GI Tract

Several studies have showcased the potential of using CNNs in different areas of digestive endoscopy. For example, when performing such examinations, the preparation and cleanliness of the GI tract are fundamental to ensure the validity of the results obtained. Nevertheless, clearly validated scales to assess this feature of endoscopy examinations are still lacking, which has inspired efforts to design AI tools based on CNN models that can automatically evaluate GI tract cleanliness in these tests [28,29]. Obviously, and in line with the advances in other areas of medicine, many studies have centred on the design of AI tools capable of detecting lesions on or alterations to the GI mucosa likely to be associated with disease [28,29,30,31], as well as specific characteristics of these changes. Indeed, the potential to apply these systems in real time could offer important benefits to the clinician, particularly when contemplating conditions that require prompt diagnosis and treatment. Moreover, these systems could potentially be used in combination or in conjunction with other AI tools, such as those designed to assess the quality of preparation, or in attempts to not only identify lesions but to also establish their malignant potential [26,32]. We must also consider that the implementation of AI tools for healthcare administration is likely to have a direct effect on gastroenterology, as it will on other clinical areas. Thus, in light of the increase in the number of AI applications being generated that may potentially be integrated into standard healthcare, it becomes more urgent to address the bioethical issues that surround their use before they are implemented in clinical practice. In this sense, it is important to note that while existing frameworks could be adjusted to regulate the use of clinical AI applications, their disruptive nature makes it more likely that new ‘purpose-built’ regulatory frameworks and guidelines should be drawn up from which regulations can be defined. Moreover, in this process, it will be important to ensure that the AI innovations they are designed to control are enhanced and not limited by the regulations drawn up.

## 2. The Emergence of AI Tools and the Questions They Raise

**Figure 1:**
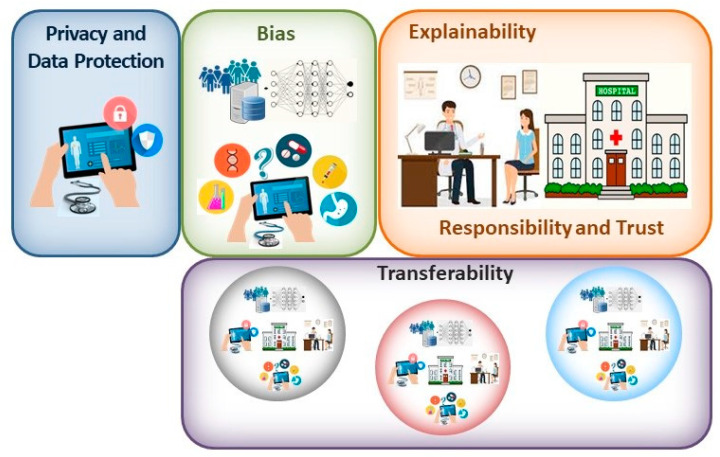
When contemplating the main bioethical issues associated with the clinical implementation of AI solutions, the principal concerns may be related to the privacy and protection of patient data; bias introduced in the design and utilization of these systems; the explainability of the tools employed; responsibility for the output and patient trust in their clinician; and finally, the transferability of these systems.

The potential benefits that are provided by any new technology must be weighed up against any risks associated with its introduction. Accordingly, if the AI tools that are developed to be used with CE are to fulfil their potential, they must offer guarantees against significant risks, perhaps the most important of which are related to issues of privacy and data protection, unintentional bias in the data and design of the tools, transferability, explainability and responsibility (Figure 1). In addition, it is clear that this is a disruptive technology that will require regulatory guidelines to be put in place to legislate the appropriate use of these tools, guidelines that are on the whole yet to be established. However, it is clear that the need for such regulation has not escaped the healthcare regulators, and, as in other fields, initiatives have been launched to explore the legal aspects surrounding the use of AI tools in healthcare that will clearly be relevant to digestive medicine as well [33,34].

### 2.1. Privacy and Data Management for AI-Based Tools

Ensuring the privacy of medical information is increasingly challenging in the digital age. Not only are electronic data easily reproduced, but they are also vulnerable to remote access and manipulation, with economic incentives intensifying cyberattacks on health-related organisations [35]. Breaches of medical confidentiality can have important consequences for patients. Indeed, they may not only be responsible for the shaming or alienation of patients with certain illnesses, but they could even perhaps limit their employment opportunities or affect their health insurance costs. As medical AI applications become more common, and as more data are collected and used/shared more widely, the threat to privacy increases. The hope is that measures such as de-identification will help maintain privacy and will require this process to be adopted more generally in many areas of life. However, the inconvenience associated with these approaches makes this unlikely to occur. Moreover, re-identification of de-identified data is surprisingly easy [36], and thus, we must perhaps accept that introducing clinical AI applications will compromise our privacy a little. This would be more acceptable if all individuals had the same chance of benefitting from these tools, in the absence of any bias, but at present, this does not appear to be the case (see below). While some progress in personal data protection has been made (e.g., General Data Protection Regulation 2016/79 in the E.U. or the Health Insurance Portability and Accountability Act in the USA: [37,38]), further advances with stakeholders are required to specifically address the data privacy issues associated with the deployment of AI applications [39].

The main aim of novel healthcare interventions and technologies is to reduce morbidity and mortality, or to achieve similar health outcomes more efficiently or economically. The evidence favouring the implementation of AI systems in healthcare generally focuses on their relative accuracy compared to gold standards [40], and as such, there have been fewer clinical trials carried out that measure their effects on outcomes [41,42]. This emphasis on accuracy may potentially lead to overdiagnosis [43]; although, this is a phenomenon that may be compensated for by considering other pathological, genomic and clinical data. Hence, it may be necessary to use more extended personal data from EHRs in AI applications to ensure the benefits of the tools are fully reaped and that they do not mislead physicians. One of the advantages of using such algorithms is that they might identify patterns and characteristics that are difficult for the human observer to perceive, and even those that may not currently be included in epidemiological studies, further enhancing diagnostic precision. However, this situation will create important demands on data management, on the safe and secure use of personal information and regarding consent for its use, accentuated by the large amount of quality data required to train and validate DL tools. Traditional opt-in/opt-out models of consent will be difficult to implement on the scale of these data and in such a dynamic environment [44]. Thus, addressing data-related issues will be fundamental to ensure a problem-free incorporation of AI tools into healthcare (Figure 1), perhaps requiring novel approaches to data protection.

One possible solution to the question of privacy and data management may come through the emergence of blockchain technologies in healthcare environments. In this sense, recent initiatives into the use of blockchain technology in healthcare may offer possible solutions to some of the problems regarding data handling and management, not least as this technology will facilitate the safer, traceable and efficient handling of an individual’s clinical information [45]. Indeed, the uniqueness of blockchain technology resides in the fact that it permits a massive, secure and decentralized public store of ordered records or events to be established [46]. Indeed, the local storage of medical information is a barrier to sharing this information, as well as potentially compromising its security. Blockchain technology enables data to be carefully protected and safely stored, assuring their immutability [47]. Thus, blockchain technology could help overcome the current fragmentation of a patient’s medical records, potentially benefitting the patient and healthcare professionals alike. Indeed, it could promote communication between healthcare professionals both at the same and perhaps at a different centre, radically reducing the costs associated with sharing medical data [48]. AI applications can benefit from different features of the use of a blockchain, offering trustworthiness, enhanced privacy and traceability. Indeed, when the data used in AI applications (both for training and in general) are acquired from a reliable, secure and trusted platform, AI algorithms will perform better.

### 2.2. The Issue of Bias in AI Applications

Among the most important issues faced by AI applications are those of bias and transferability [49]. Bias may be introduced through the training data employed or by decisions that are made during the design process [42,50,51,52]. In essence, ML systems are shaped by the data on which they are trained and validated, identifying patterns in large datasets that reproduce desired outcomes. Indeed, AI systems are tailor-made, and as such, they are only as good as the data with which they are trained. As such, when these data are incomplete, unrepresentative or poorly interpreted, the end result can be catastrophic [53,54]. One specific type of bias, spectrum bias, occurs when a diagnostic test is studied in individuals who differ from the population for which the test was intended. Indeed, spectrum bias has been recognized as a potential pitfall for AI applications in capsule endoscopy (CE) [19], as well as in the field of cardiovascular medicine [55]. Hence, AI learning models might not always be fully valid and applicable to new datasets. In this context, the integration of blockchain-enabled data from other healthcare platforms could serve to augment the number of what would otherwise be underrepresented cases in a dataset, thereby improving the training of the AI application and ultimately, its successful implementation.

**Figure 2:**
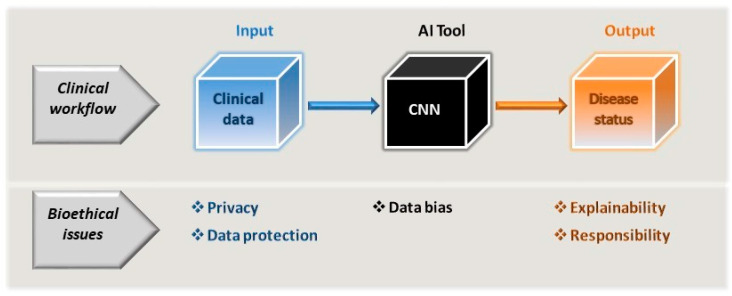
As part of the clinician’s workflow and decision-making process, the AI tools driven by CNNs can be considered a black box subject to data bias. As such, the AI tool itself cannot be allowed to introduce bias through its very design or to exacerbate any bias inherent to the input data used. The model input is essentially the patient’s clinical (or clinically related) data, which is subject to the constraints of privacy and data protection. As a consequence of using the tool, the clinician will extract information regarding the patient’s disease status and they must be in a position to be able to accept and explain the output of the model, and along with the healthcare providers, accept the same level of responsibility for this as would be expected in any clinical workflow.

In real life, any inherent bias in clinical tools cannot be ignored and must be considered before validating AI applications. As a result, overfitting of these models should not be ignored, a phenomenon that occurs when the model is too tightly tuned to the training data, and as a result, it does not function correctly when fed with other data [56]. This can be avoided by using larger datasets for training and by not training the applications excessively, and possibly also by simplifying the models themselves. The way outcomes are identified is also entirely dependent on the data the models are fed. Indeed, there are examples of different pathologies where certain physical characteristics achieve better diagnostic performance, such as lighter rather than darker skin, yet perhaps this is a population that is overrepresented in the training data. Consequently, it is possible that only those with fair skin will fully benefit from such tools [57,58]. Human decisions may also skew AI tools, such that they may act in discriminatory ways [54]. Disadvantaged groups may not be well-represented in the formative stages of evidence-based medicine [59], and unless rectified, and human interventions can combat this bias, it will almost certainly be carried over into AI tools. Hence, programmes will need to be established to ensure ethical AI development, such as those contemplated to detect and eliminate bias in data and algorithms [60,61]. While bias may emerge from poor data collection and evaluation, it can also emerge in systems trained on high-quality datasets. Aggregation bias can emerge from using a single population to design a model that is not optimal for another group [49,53]. Thus, the potential that bias exists must be faced and not ignored, searching for solutions to overcome this problem rather than rejecting the implementation of AI tools on this basis (Figure 1 and Figure 2).

In association with bias, transferability to other settings is a related and significant issue for AI tools [62]. An algorithm trained and tested in one environment will not necessarily perform as well in another environment, and it may need to be retrained on data from the new environment. Even so, transferability is not ensured, and hence, AI tools must be carefully designed, tested and evaluated in each new context prior to their use with patients [63]. This issue also implies there must be significant transparency about the data sources used in the design and development of these systems, with the ensuing demands on data protection and safety.

### 2.3. The Explainability, Responsibility and the Role of the Clinician in the Era of AI-Based Medicine

Another critical issue with regards to the application of DL algorithms is that of explainability (Figure 2; [64,65]) and interpretability [41,42,50,66]. When explainable, what an algorithm does and the value it encodes can be readily understood [67]. However, it appears that less explainable algorithms may be more accurate [53,68], and thus, it remains unclear if it is possible to achieve both these features at the same time. How algorithms achieve a particular classification or recommendation may even be unclear to some extent to designers and users alike, not least due to the influence of training on the output of the algorithms and that of user interactions. Indeed, in situations where algorithms are being used to address relatively complex medical situations and relationships, this can lead to what is referred to as “black-box medicine”: circumstances in which the basis for clinical decision making becomes less clear [69]. While the explanations a clinician may give for their decisions may not be perfect, they are responsible for these decisions and can usually offer a coherent explanation if necessary. Thus, should AI tools be allowed to make diagnostic, prognostic and management decisions that cannot be explained by a physician [64,65]? Some lack of explainability has been widely accepted in modern medicine, with clinicians prescribing aspirin as an analgesic without understanding its mechanism of action for nearly a century [70]. Moreover, it still remains unclear why Lithium acts as a mood stabilizer [70]. If drugs can be prescribed without understanding how they work, then can we not use AI without fully understanding how it reaches a decision? Yet as we move towards greater patient inclusion in their healthcare decisions, the inability of a clinician to fully explain decisions based on AI may become more problematic. Hence, perhaps we are right to seek systems that allow us to trace how conclusions are reached. Moreover, only through some degree of knowledge of AI can physicians be aware of what these tools can actually achieve and when they may be performing irregularly.

AI is commonly considered to be of neutral value, neither intrinsically good nor bad, yet it is capable of producing good and bad outcomes. AI algorithms explicitly or implicitly encode values as part of their design [71,72], and these values inevitably influence patient outcomes. For example, algorithms will often be designed to prioritise a false-negative rather than false-positive identification, or to perform distinctly depending on the quality of the preparation. While the performance of AI systems would represent a limiting factor for diagnostic success, additional factors will also influence their accuracy and sensitivity, such as the data on which they are trained, how the data are used by the algorithm, and any conscious or unconscious biases that may be introduced. Indeed, the digitalisation of medicine has been said to have shifted the physician’s attention away from the body towards the patient’s data [53,73], and the introduction of AI tools runs the risk of further exacerbating this movement.

Introducing AI tools into medicine also has implications for the allocation of responsibility regarding treatment decisions (Figure 2) and any adverse outcomes based on the use of such tools, as discussed in greater depth elsewhere [53]. At present, there appears to be a void regarding legal responsibility if the use of AI applications produces harm [74], and there are difficulties in clearly establishing the autonomy and agency of AI [75]. Should any adverse event occur, it is necessary to establish if any party failed in their duty or if errors occurred, attributing responsibility accordingly. Responsibility for the use of the AI will usually be shared between the physician and institution where the treatment was provided, but what of the designers? Responsibility for acting on the basis of the output of the AI will rest with the physician, yet perhaps no party has acted improperly or the AI tool behaved in an unanticipated manner. Indeed, if the machine performs its tasks reliably, there may be no wrongdoing even when it fails. The points in an algorithm at which decisions are made may be complicated to define, and thus, clinicians may be asked to take responsibility for decisions they have not made when using a system that incorporates AI. Importantly, this uncertainty regarding responsibility may influence the trust of a patient in their clinician [76]. Accordingly, the more that clinicians and patients rely upon clinical AI systems, the more that trust may shift away from clinicians toward the AI tools themselves [53].

In relation to the above, the implementation of AI tools may also raise concerns about the role of clinicians. While there are fears that they will be ‘replaced’ by AI tools [77], the ideal situation would be to take advantage of the strengths of both humans and machines. AI applications could help to compensate for shortages in personnel [78], they could free up more of a clinicians’ time, enabling them to dedicate this time to their patients or other tasks [62], or they might enhance the clinician’s capacity in terms of the number of patients they could treat. While decision making in conjunction with AI should involve clinicians, the issue of machine–human disagreement must be addressed [42,52]. Alternatively, should we be looking for opportunities to introduce fully automated clinical AI solutions? For example, could negative results following AI-based assessment of GI examinations be communicated directly to the patient? While this might be more efficient, it brings into question the individual’s relationship with the clinician. Indeed, the dehumanisation of healthcare may have a detrimental rather than a beneficial effect given the therapeutic value of human contact, attention and empathy [79,80]. While clinicians may have more time to dedicate to their patients as more automated systems are incorporated into their workflow, they may be less capable to explain AI-based healthcare decision making [51]. Moreover, continued use of AI tools could deteriorate a clinician’s skills, a phenomenon referred to as “de-skilling” [67], such as their capacity to interpret endoscopy images or to identify less obvious alterations. Conversely, automating workflows may expose clinicians to more images, honing their skills by greater exposure to clinically relevant images, yet maybe at the cost of seeing fewer normal images. In addition, more extended use of automated algorithms may lead to a propensity to accept automated decisions even when they are wrong [62,81,82], with a negative effect on the clinician’s diagnostic precision. Thus, efforts must be made to ensure that the clinician’s professional capacity remains fine-tuned to avoid generating a dependence on automated systems [41,50,81,83] and to avoid any potential loss of skills (e.g., in performing and interpreting endoscopies) when physicians are no longer required to use (the phenomenon of de-skilling has also been dealt with in more detail elsewhere [53,67]).

**Figure 3:**
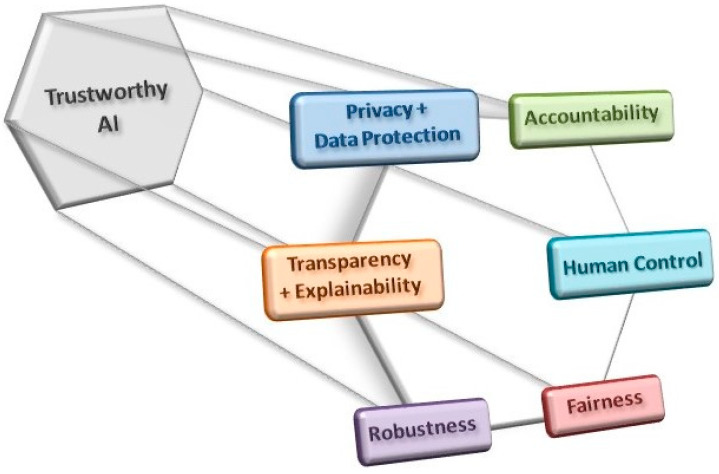
The use and development of AI tools must comply with a series of criteria in order to obey ethical guidelines and good practices in their implementation, all with a view to establishing trustworthy AI applications.

Other issues have been raised in association with the clinical introduction of AI applications, such as whether they will lead to greater surveillance of populations and how this should be controlled. Surveillance might compromise privacy but it could also be beneficial, enhancing the data with which the DL applications are trained, so perhaps this is an issue that will be necessary to contemplate in regulatory guidelines. Another issue that also needs to be addressed is the extent to which non-medical specialists such as computer scientists and IT specialists will gain power in clinical settings. Finally, the fragility associated with reliance on AI systems and the potential that monopolies will be established in specific areas of healthcare will also have to be considered [53]. In summary, it will be important to respect a series of criteria when designing and implementing AI-based clinical solutions to ensure that they are trustworthy (Figure 3; [84]).

## 3. The Bright Side and Benefits of AI in the Clinic

We are clearly at an interesting moment in the history of medicine as we embrace the use of AI and big data as a further step in the era of medical digitalisation. Despite the many challenges that must be faced, this is clearly going to be a disruptive technology in many medical fields, affecting clinical decision making and the doctor–patient dynamic in what will almost certainly be a tremendously positive way. Different levels of automation can be achieved by introducing AI tools into clinical decision-making routines, selecting between fully automated procedures and aids to conventional protocols as specific situations demand. Some issues that must be addressed prior to the clinical implementation of AI tools have already been recognised in healthcare scenarios. For example, bias is an existing problem evident through inequalities in the care received by some populations. AI applications can be used to incorporate and examine large amounts of data, allowing inequalities to be identified and leveraging this technology to address these problems. Through training on different populations, it may be possible to identify specific features of these populations that have an influence on disease prevalence, and/or on its progression and prognosis. Indeed, the identification of population-specific features that are associated with disease will undoubtedly have an important impact on medical research. However, there are other challenges that are posed by these systems that have not been faced previously and that will have to be resolved prior to their widespread incorporation into clinical decision decision-making procedures [85].

Automating procedures is commonly considered to be associated with greater efficiency, reduced costs and savings in time. The growing use of CE in digestive healthcare and the adaptation of these systems to an increasing number of circumstances generates a large amount of information and each examination may require over an hour to analyse. This not only requires the dedication of a clinician or specialist, and their training, but it may increase the chance of errors due to tiredness or monotony [86] (not least as lesions may only be present in a small number of the tens of thousands of images obtained [24]). DL tools have been developed based on CNNs to be used in conjunction with different CE techniques that aim to detect lesions or abnormalities in the intestinal mucosa [27,30,32,87,88]. These algorithms are capable of reducing the time required to read these examinations to a question of minutes (depending on the computational infrastructures available). Moreover, they have been shown to be capable of achieving accuracies and results not dissimilar to the current gold standard (expert clinician visual analysis), performances that will most likely improve with time and use. In addition, some of these tools will clearly be able to be used in real time, with the advantages that this will offer to clinicians and patients alike [89]. As well as the savings in time and effort that can be achieved by implementing AI tools, these advances may to some extent also drive the democratization of medicine and help in the application of specialist tools in less well-developed areas. Consequently, the use of AI solutions might reduce the need for specialist training to be able to offer healthcare services in environments that may be more poorly equipped. This may represent an important complement to systems such as CE that involve the use of more portable apparatus capable of being used in areas with more limited access and where patients may not necessarily have access to major medical facilities. Indeed, it may even be possible to use CE in the patient’s home environment.

It should also be noted that enhancing the capacity to review and evaluate large numbers of images in a significantly shorter period of time may also offer important benefits in the field of clinical research. Drug discovery programmes and research into other clinical applications are notoriously slow and laborious. Thus, any tools that can help speed up the testing and screening capacities in research pipelines may have important consequences in the development of novel treatments. Moreover, when performing multicentre trials, the variation in the protocols implemented is often an additional and undesired variable. Hence, medical research and clinical trials in particular will benefit from the use of more standardized and less subjective tools. Accordingly, offering researchers the ability to access large amounts of data that have been collected in a uniform manner, even when obtained from different sites, and making it possible to perform medical examinations more swiftly, can only benefit clinical research studies and trials.

## 4. Concluding Remarks

In terms of the introduction of AI applications into clinical pipelines, we consider the future to be one of great promise. While it is clear that it will not be seamless and it will require the coordinated effort of many stakeholders, the pot of gold that awaits at the end of the rainbow seems to be getting ever bigger. These applications raise important bioethical issues, not least those related to privacy, data protection, data bias, explainability and responsibility. Consequently, the design and implementation of these tools will need to respect specific criteria to ensure that they are trustworthy ([84]). Since these are tools that are breaking new ground, the solutions to these issues may also need to be defined ad hoc, adopting novel procedures. This is an issue that cannot be overlooked as it may be critical to ensure that the opportunities offered by this technology do not slip through our hands.

## Data Availability

Not applicable.
